# Vaccine Hesitancy Drives Low Human Papillomavirus Vaccination Coverage in Girls Attending Public Schools in South Africa

**DOI:** 10.3389/fpubh.2022.860809

**Published:** 2022-05-24

**Authors:** Languta A. Khosa, Johanna C. Meyer, Feni M. M. Motshwane, Carine Dochez, Rosemary J. Burnett

**Affiliations:** ^1^Department of Public Health Pharmacy and Management, Sefako Makgatho Health Sciences University, Pretoria, South Africa; ^2^South African Vaccination and Immunisation Centre, Sefako Makgatho Health Sciences University, Pretoria, South Africa; ^3^Child, Youth and School Health Cluster, Integrated School Health Programme, National Department of Health, Pretoria, South Africa; ^4^Network for Education and Support in Immunisation, Department of Family Medicine and Population Health, University of Antwerp, Antwerp, Belgium; ^5^Department of Virology, Sefako Makgatho Health Sciences University, Pretoria, South Africa

**Keywords:** vaccine hesitancy, South Africa, human papillomavirus vaccine, vaccination coverage, public sector schools, 5C scale

## Abstract

Girls aged ≥9 years attending South African public sector schools are provided with free human papillomavirus (HPV) vaccination, through a schools-based programme. HPV vaccine misinformation spread *via* social media in 2014, was identified as a barrier to obtaining parental informed consent in some districts, including Sedibeng District, which subsequently had the lowest HPV vaccination coverage in Gauteng Province in 2018. This study investigated vaccine hesitancy in caregivers of girls in Grade 4 to 7 aged ≥9 years attending public schools in Sedibeng District. A cross-sectional survey using a self-administered questionnaire was conducted among caregivers of age-eligible girls attending all public schools in Sedibeng District with first dose HPV vaccination coverage of <70%. The questionnaire included demographics; HPV vaccination status of girls; reasons for not being vaccinated; and a 5-item tool measuring the determinants of vaccine hesitancy (5C scale), using a 7-point Likert scale. Data were coded and captured on Microsoft Excel®. Except for collective responsibility which was reverse scored, the other 5C items (confidence, complacency, constraints, and calculation) were captured as follows: 1 = strongly disagree, 2 = moderately disagree, 3 = slightly disagree, 4 = neutral, 5 = slightly agree, 6 = moderately agree and 7 = strongly agree. Descriptive and inferential statistical analyses were conducted using Epi Info^TM^. Of the principals of all schools with <70% HPV vaccination coverage, 69.6% (32/46) gave permission. The response rate from caregivers of girls present on the day of data collection was 36.8% (1,782/4,838), with 67.1% (1,196/1,782) of respondents reporting that their daughters had received ≥1 dose of HPV vaccine. Only 63.1% (370/586) of respondents with unvaccinated daughters answered the question on reasons, with 49.2% (182/370) reporting reasons related to vaccine hesitancy. Statistically significant differences between caregivers of vaccinated and unvaccinated daughters were identified for four of the five determinants of vaccine hesitancy: confidence (vaccinated group higher), complacency (unvaccinated group higher), constraints (unvaccinated group higher) and collective responsibility (vaccinated group higher). This is the first South African study to (a) report results of the 5C scale, which was found to be very useful for predicting vaccination uptake; and (b) confirm that the relatively low HPV vaccination coverage in Sedibeng District is largely driven by reasons related to vaccine hesitancy.

## Introduction

South Africa is home to <1% of the global population of women aged ≥15 years, yet bears 2.3% and 1.8% of global new cervical cancer cases and deaths from cervical cancer respectively (1). This high burden of cervical cancer prompted the South African National Department of Health to introduce free human papillomavirus (HPV) vaccination for all public sector school girls aged ≥9 years in 2014 (2, 3). Public schools targeted by this programme include ordinary primary schools, intermediary schools, combined schools and special schools (i.e. school providing education for learners with special needs) (3). This schools-based HPV vaccination programme is offered in partnership with the Department of Basic Education (DBE), through South Africa's Integrated School Health Programme (ISHP). It is based on a two-dose schedule of the bivalent HPV vaccine given six months apart, delivered through campaigns held twice a year (2, 3). Originally girls in Grade 4 were targeted, but in 2020 this was changed to girls in Grade 5 (https://www.westerncape.gov.za/general-publication/hpv-vaccinations), as many girls in Grade 4 were found to be younger than 9 years, and therefore missed their vaccinations. While first dose HPV vaccination coverage in 2014 was relatively high at 86.6% (3), since then there has been a steady decline in coverage, with two dose coverages of 65% in 2014; 61% in 2015 and 2016; and 56% in 2017, 2018 and 2019 being reported by the World Health Organization (WHO) and United Nations Children's Fund, annual Joint Reporting Form (https://bit.ly/3nHAQXI).

At the time when the first HPV vaccination campaign was launched in 2014, HPV vaccine misinformation spread *via* social media was identified as a barrier to obtaining parental informed consent in some areas of South Africa (3). In particular, vaccine safety misinformation spread by anti-vaccination groups in high-income countries, dominated the social media conversation in South Africa (3). This finding is supported by an earlier study on the South African Internet, conducted from 2011 to 2013, which reported that 77.6% of anti-vaccination claims originated from websites within the United States of America (USA) (4). Links to these websites have gone viral globally through social media, the use of which has been identified as a strong predictor of vaccine hesitancy (5), defined by the WHO in 2014 as a “delay in acceptance or refusal of vaccination despite availability of vaccination services” (6). The viral spread of vaccine misinformation and disinformation was identified in 2018 as a global threat to public health (7). While the extent to which social media misinformation has contributed to the steady decline in HPV vaccination coverage of South African public sector school girls is unknown, it is highly likely to have played a role in creating HPV vaccine hesitancy amongst South African caregivers of these girls. This is supported by a study reporting that a third of public comments made in response to an HPV vaccination-related Facebook post by the Western Cape Department of Health in 2019, were either vaccine hesitant or made by vocal vaccine deniers (8).

One of the districts reportedly affected by anti-vaccination lobbying on social media during the HPV vaccination programme roll-out in 2014, was Sedibeng District in Gauteng Province (personal communication, ISHP). Also, Sedibeng District had the lowest HPV vaccination coverage in Gauteng Province in 2018 (personal communication, ISHP). This study aimed to investigate vaccine hesitancy in caregivers of girls in Grades 4 to 7 aged ≥9 years attending public schools in the Sedibeng District of Gauteng. Objectives included investigating reasons why girls had not been vaccinated, and measuring and comparing levels of determinants of vaccine hesitancy between caregivers of vaccinated and unvaccinated girls.

## Methods

### Study Population and Study Design

The Sedibeng District is comprised of three local municipalities: Emfuleni, Lesedi and Midvaal Local Municipalities (https://municipalities.co.za/overview/114/sedibeng-district-municipality). In 2019 there were 156 public sector primary schools in the Sedibeng District, of which 46 had HPV vaccination first dose coverage below 70% in 2018 (personal communication, ISHP). All 46 schools were targeted by the survey, which was conducted from 26 August to 20 September 2019. This cross-sectional survey utilized a questionnaire which was self-administered by caregivers of girls in Grades 4 to 7 aged ≥9 years, attending the schools where permission had been granted by the school principals.

### Data Collection Tool

The questionnaire started with the title, followed by a message of gratitude for participation, details on the length of the questionnaire (in pages and time taken to complete), the contact details of the researcher should further clarity be required, the aim and objectives of the survey, and ended with a consent statement. Thereafter there were sections on demographics of both caregivers and girls; the HPV vaccination status of the girls; a question on reasons for the girl not being vaccinated, with 15 selection options, the last option being “other (please specify)”; and a 5-item tool for measuring the determinants of vaccine hesitancy, based on the 5C model developed and proposed by Betsch et al. (9). Participants had to select their level of agreement with the 5 statements, using a 7-point Likert scale (strongly disagree, moderately disagree, slightly disagree, neutral, slightly agree, moderately agree, and strongly agree). The questionnaire was made available in English, Sesotho and Afrikaans (the most common languages spoken in the Sedibeng District) depending on the home language of the girls' caregivers. The questionnaire was first compiled in English and thereafter translated to Sesotho and Afrikaans by two independent translators, one fluent in Sesotho and English and one fluent in Afrikaans and English. The final data collection tool is available in Supplementary Data Sheet 1.

#### Rationale for Using the 5C Model

When the WHO first defined vaccine hesitancy in 2014, they had built upon earlier work identifying the key drivers of vaccine hesitancy as confidence (trust in vaccine safety and effectiveness, in the national health authorities funding the vaccination programme, and in local healthcare providers delivering vaccination services); complacency (perception of low risk from vaccine-preventable disease [VPD]) and convenience (barriers to vaccination including ease of accessing information about vaccination, and accessing vaccination services), known as the “3Cs model” (6). Most vaccine hesitancy studies have concentrated on measuring vaccine confidence as a predictor of vaccination uptake (9), and it is mainly vaccine confidence that gets eroded by misinformation/disinformation spread by social media (7).

It has been argued that the term “vaccine hesitant” is a misnomer for a parent whose child is unvaccinated because of practical barriers to vaccination (9, 10). In the 3C model, convenience is the umbrella term for these lack of access issues, related to the ease of accessing information about vaccination and accessing vaccination services. Since the term “convenience” seems to confine the construct to decisions made by individuals, whereas many access barriers are related to the health system (9, 10), the alternative term “constraints” was proposed in 2018 as part of the 5C model (9). The 5C model defines confidence, complacency, constraints, calculation (the degree to which individuals search for information in order to weigh the risks of the VPD against the risks of being vaccinated against the VPD) and collective responsibility (willingness to vaccinate oneself in order to protect others through herd immunity) as the “psychological antecedents of vaccination” (9).

The 5C model has subsequently become an important tool for measuring the determinants of vaccine hesitancy, and has been used for this purpose on various populations in different countries, including Hong Kong nurses (11); USA patients (12); German physicians (13, 14); Bangladeshi adults (15); Swiss university students (16); and adults in Egypt, United Arab Emirates and Jordan (17). Although South Africa is yet to validate a tool for measuring the extent and determinants of vaccine hesitancy, there is evidence from South Africa that the 5C tool may be appropriate for investigating HPV vaccine hesitancy in this setting (8). The results of this study will provide important information on the suitability of the 5C model for the South African setting, and help to identify further adaptations that may be necessary before validation is attempted.

### Data Collection

#### Data Collection Team and Training

The first author trained a team of four research assistants (three masters students and one public health professional) to assist with (a) obtaining permission from school principals; (b) conducting information sessions with class teachers and learners; and (c) recording the numbers of surveys distributed to, and collected from each class at each school.

#### Data Collection Process

Data collection commenced after obtaining (a) ethical clearance from the Sefako Makgatho University Research Ethics Committee (SMUREC/P/104/2019:PG), (b) permission from the Gauteng Department of Education and Sedibeng District Department of Education, and (c) permission from the individual school principals. Data were collected from 26 August to 20 September 2019. The questionnaire included a consent statement as well as the ethics clearance number. At each of the schools where the principal had given permission for data collection, a team member had sessions with the class teachers and female learners, where they explained the purpose of the survey, and the importance of giving the questionnaires to their caregivers, and returning them back to class.

Self-administered questionnaires in the language of choice, were handed out in sealed envelopes to female learners in Grades 4 to 7 by a team member, who kept a record of the number of envelopes distributed per class in each school. The sealed envelope also contained an empty envelope in which to place the completed questionnaire for the learner to bring back to school. A sealed box with a posting slot for this purpose was placed in each classroom. Teachers were requested to remind the girls about returning the questionnaires daily, until the boxes were collected the following week by a team member, who counted and recorded the numbers of returned envelopes for each class.

#### Data Capture and Cleaning

The raw data from the questionnaires were coded and captured on a Microsoft Excel® 2016 (Microsoft Office, USA) spreadsheet. The free text that respondents provided for the “other” reason option for not being vaccinated, was captured verbatim. Except for collective responsibility which was reverse scored, the 5C items were captured as follows: 1 = strongly disagree, 2 = moderately disagree, 3 = slightly disagree, 4 = neutral, 5 = slightly agree, 6 = moderately agree and 7 = strongly agree. Data were checked to ensure reliability of data entry. The checked spreadsheet was imported into Microsoft Access® 2016 (Microsoft Office, USA) for cleaning. Access® queries were run to identify records containing inconsistent data, and where these were found (e.g.: records where reasons for not being vaccinated were given for girls who were vaccinated), the relevant questionnaire was reviewed and any data capture errors were corrected.

### Pilot Study

The questionnaire was pre-tested in a pilot study conducted in one of the primary schools in the Sedibeng District, which had >70% HPV vaccination coverage and thus was not included in the study. Pre-testing aimed to ascertain if the questions were well-comprehended and appropriate for eliciting the required information. Furthermore, piloting the study provided an overview of the actual study on a small scale, which assisted in making the necessary changes to the data collection instrument and the data collection process, prior to the commencement of the actual study. After the pilot study was conducted, necessary amendments and additions were made to the data collection instrument and process. The final data collection tool is available in Supplementary Data Sheet 1.

### Data Analysis

Free text reasons were analyzed and coded by the first author, who created new codes for reasons that did not fit with the original list of reasons options. These codes were then checked by one of the research assistants, with discrepancies being checked by an independent researcher working within the over-arching project, who made the final decision and validated all the coding. A category for reasons related to vaccine hesitancy was created, based on the WHO definition of a “delay in acceptance or refusal of vaccination despite availability of vaccination services” (6). Data were imported into Epi Info™ version 7.2.4.0 (Centers for Disease Control and Prevention, USA) for descriptive and inferential statistical analysis. Descriptive data analysis included calculating the frequency distribution of categorical data (demographic data except for age, HPV vaccination status of daughters, reasons for not being vaccinated); and calculating measures of central tendency (mean; median) and dispersion (range; standard deviation [SD]) for continuous data (age; 5C item scores). Inferential statistical analysis included the independent *t*-test for measuring the statistical significance of the differences between the means of the 5C scores of caregivers of vaccinated and unvaccinated girls. *P*-values < 0.05 were considered statistically significant.

## Results

### Pilot Study Results

The pilot study was conducted on 67 volunteers. The maximum time taken to complete the questionnaire was 10 min. During piloting the principal advised that the majority of the learners spoke Afrikaans as their home language, thus the need for translating the data collection tool into Afrikaans was identified. No further changes were made to the data collection tool or data collection process.

### Response Rate

Of all schools requested to participate in the study, permission was denied by 30.4% (14/46). The reasons given by the 14 principals for denying permission included (a) the survey was being conducted at a very busy time in the school calendar (4 principals); (b) the perception that the majority of caregivers are opposed to HPV vaccination, thus the principal did not want to upset them (3 principals); (c) no reasons given (7 principals, 2 of whom chased the research team off the premises). Thus permission to conduct the study was granted for 69.6% (32/46) of the schools. Of these 32 schools, all girls in Grades 4 to 7 present on the day of data collection (4,838) were handed questionnaires to take home for their caregivers to complete. In total, 63.9% (3,091/4,838) of questionnaires were returned. Of these 17.9% (553/3,091) were returned without any questions being answered, with 24.8% (137/553) of these being over-written with a statement indicating that the respondents do not give consent for HPV vaccination, i.e. these respondents apparently mistook the questionnaire for a consent form for HPV vaccination. Of the remaining questionnaires, the question on HPV vaccination status of the girls was answered by 1,784 respondents, 2 of whom had daughters too young to be vaccinated (i.e. <9 years old). Thus the final sample consisted of 1,782, giving a response rate of 36.8% (1,782/4,838).

### Socio-Demographics

#### Caregivers

Data on age of caregiver was provided by 1,472 respondents, with the median age being 38.0 years and the mean being 40.5 years (SD: 10.4; range: 19.0–98.0). The majority were aged between 30 and 49 years (62.4% [1,112/1,472]) and were the biological parents of the girls (83.7% [1,476/1,763]). The demographic characteristics of the respondents are detailed in Table 1.

**Table 1:** Frequency distributions of socio-demographics of caregivers.

**Characteristics**		**n (%)**
Sex (*n* = 1,765)	Female	1,627 (92.2)
	Male	138 (7.8)
Race (*n* = 1,725)	African	1,414 (82.0)
	White	290 (16.8)
	Colored	17 (1.0)
	Asian	4 (0.2)
Religion (*n* = 804)	Christian	795 (98.9)
	African Traditional	5 (0.6)
	Ancestral	1 (0.1)
	Hindu	2 (0.3)
	Rastafarian	1 (0.1)
Relationship with child (*n* = 1,763)	Mother	1,350 (76.6)
	Father	126 (7.2)
	Grandparent	188 (10.7)
	Sibling	44 (2.5)
	Aunt	33 (1.9)
	Uncle	9 (0.5)
	Foster parent/guardian	13 (0.7)
Marital status (*n* = 1,688)	Single	533 (31.6)
	Married	613 (36.3)
	Widowed	118 (7.0)
	Divorced	109 (6.5)
	Living with partner	248 (14.7)
	*Lobola	67 (4.0)
Educational level (*n* = 1,612)	No education	40 (2.5)
	Some primary education	104 (6.5)
	Primary education	124 (7.7)
	Some secondary education	353 (21.9)
	Secondary education	580 (36.0)
	Tertiary education	411 (25.5)
Employment status (*n* = 1,578)	Permanently employed	509 (32.3)
	Self-employed	202 (12.8)
	Unemployed	813 (51.5)
	Retired	26 (1.7)
	Disabled, unable to work	28 (1.8)

* *Lobola = Traditional marriage in which a “bride price” has been paid*.

#### Girls Age and Eligibility for HPV Vaccination

Data on date of birth of girls was provided by 1,686 respondents, with the median age being 11.4 years and the mean being 11.5 years (SD: 1.3; range: 9–19). Of the 96 girls for whom the respondents had not provided a date of birth, 66.7% (64/96) had received ≥1 dose of HPV vaccine, thus these girls must have been aged ≥9 years. None of the 32 respondents who did not provide a date of birth and whose daughters were not vaccinated, gave the reason “my daughter is too young.” It was thus assumed that all 32 girls without a date of birth were age-eligible for HPV vaccination.

#### Girls Demographics Other Than Age

The majority of girls were African (82.6% [1,455/1,762]), with 16.0% (282/1,762) being white, 1.2% (21/1,762) being of mixed descent and 0.2% (4/1,762) being Asian. The majority (58.0% [1,034/1,782]) of girls were in Grades 4 and 5. Table 2 provides further details of the girls' grades stratified by their vaccination status.

**Table 2:** Frequency distribution of girls' grades stratified by HPV vaccination status (*n* = 1,782).

	**Vaccinated with ≥1 dose**	**Unvaccinated**	**Total**
	n (%)^*^	n (%)^*^	**n (%)**
Grade 4	354 (64.7)	193 (35.3)	547 (30.7)
Grade 5	303 (62.2)	184 (37.8)	487 (27.3)
Grade 6	317 (72.4)	121 (27.6)	438 (24.6)
Grade 7	222 (71.6)	88 (28.4)	310 (17.4)
Totals	1,196 (67.1)	586 (32.9)	1,782 (100)

* *Denominators are the totals in each grade*.

### HPV Vaccination Coverage

The daughters of 67.1% (1,196/1,782) of respondents had received ≥1 dose of HPV vaccine. Of respondents whose daughters were vaccinated, 1,074 answered the question on number of doses received, and of these, 58.5% (628/1,074) reported that their daughter had received both HPV vaccine doses. Of respondents who answered the question on place where vaccination was received, 90.8% (1,049/1,115) reported that their daughters had been vaccinated at school. Caregivers of vaccinated girls were also asked a question on what grade their daughter was in, at the time she was vaccinated. Of those who answered this question, 80.4% (707/879) were vaccinated when they were in Grade 4; 13.4% (118/879) when they were in Grade 5; 1.5% (13/879) when they were in Grade 6; 0.8% (7/879) when they were in Grade 7; and 3.9% (34/879) were vaccinated in two separate grades.

### Reasons Why Girls Were Not Vaccinated

Respondents were allowed to select more than one reason for their daughter being unvaccinated. Of respondents whose daughters were unvaccinated, 63.1% (370/586) answered this question, and gave a total of 654 reasons (Table 3). Of respondents reporting reasons, 49.2% (182/370) reported reasons related to vaccine hesitancy. Of all reasons reported by these respondents, reasons related to vaccine hesitancy (bold type in Table 3) made up 53.5% (350/654).

**Table 3:** Frequency distribution of reasons for non-vaccination.

	**Respondents (*****n*** **= 370)**	
**Reasons for not vaccinating**	**n**	**%**
I am not aware of a HPV vaccination programme at school	173	46.8
**I am concerned about side effects and the safety of this vaccine**	118	31.9
**Someone told me they or their child had a bad reaction to the HPV vaccine**	56	15.1
I haven't had time to think about it yet	44	11.9
**My daughter is not at risk of cervical cancer**	43	11.6
The nurses never came to the school to vaccinate the girls	41	11.1
**I do not think that the HPV vaccine is effective**	39	10.5
**My daughter has a fear of needles**	34	9.2
My daughter was absent from school on vaccination day	29	7.8
**I have no faith in vaccinations**	23	6.2
**My religion prohibits vaccination**	15	4.1
**My healthcare provider has advised me against the HPV vaccine**	13	3.5
**My daughter had a bad experience with a previous vaccination or a vaccinator**	9	2.4
My daughter was underage at the time vaccination was offered	9	2.4
My child has an illness that doesn't allow for vaccinations	8	2.2
**Total number of reasons**	**654**	

*Reasons in bold were categorized as related to vaccine hesitancy*.

Table 4 illustrates the proportion of reasons that were categorized as unrelated to vaccine hesitancy, which were given by the same respondents together with reasons related to vaccine hesitancy. Two of the reasons considered as unrelated to vaccine hesitancy, have sometimes been reported as related to vaccine hesitancy: “I haven't had time to think about it yet” (18) and “My daughter was absent from school on vaccination day” (19). Recoding these reasons only when given together with reasons related to vaccine hesitancy, as “postponing vaccination” (22 reasons) and “absent to avoid vaccination” (15 reasons) respectively, resulted in 59.2% (387/654) of reasons being related to vaccine hesitancy. Although recoding of these reasons increased the proportion of reasons related to vaccine hesitancy, the proportion of respondents who gave reasons related to vaccine hesitancy remained unchanged, as these respondents had given other reasons related to vaccine hesitancy.

**Table 4:** Frequency distribution of reasons unrelated to vaccine hesitancy given together with reasons related to vaccine hesitancy by the same respondents.

		**Vaccine hesitancy reasons by same respondents**	
**Reasons unrelated to vaccine hesitancy**	**Total**	**n**	**%**
I am not aware of a HPV vaccination programme at school	173	41	23.7
**I haven't had time to think about it yet**	44	^**(a)**^ **22**	50.0
The nurses never came to the school to vaccinate the girls	41	22	53.7
**My daughter was absent from school on vaccination day**	29	^**(b)**^ **15**	51.7
My daughter was underage when vaccination was offered	9	2	22.2
My daughter has an illness that doesn't allow for vaccinations	8	3	37.5

*Reasons in **bold** may be related to vaccine hesitancy (18, 19). Number of reasons recoded as (a) “postponing vaccination” and (b) “absent to avoid vaccination” respectively*.

Of the schools participating in the survey, 87.5% (28/32) were named by respondents who gave the reason “I am not aware of a HPV vaccination programme at school.” The number of girls at each school who were unvaccinated because of this reason, ranged from 1 to 19, with 25% (7/28) of the schools having ≥10 girls being unvaccinated because of this reason.

### 5C Results

Table 5 provides details on the frequency distributions of all responses to the 5C statements, stratified by vaccination status of daughters. There were statistically significant differences in the mean scores for four of the determinants of vaccine hesitancy: *confidence* (higher in the vaccinated group), *complacency* (higher in the unvaccinated group), *constraints* (higher in the unvaccinated group) and *collective responsibility* (higher in the vaccinated group). There was very little difference between the mean scores of the two groups for calculation. See Table 6 for further details on 5C means and *t*-test *p*-values.

**Table 5:** Frequency distributions of all responses to the 5C statements.

**Statement**	**Agreement level**	**Vaccinated** **n (%)**	**Unvaccinated** **n (%)**
**Confidence:** I am completely confident that the HPV vaccine is safe	Strongly disagree	29 (2.9)	70 (15.6)
	Moderately disagree	8 (0.8)	26 (5.8)
	Slightly disagree	11 (1.1)	18 (4)
	Neutral	90 (9.1)	84 (18.7)
	Slightly agree	48 (4.9)	31 (6.9)
	Moderately agree	202 (20.5)	63 (14.0)
	Strongly agree	600 (60.7)	158 (35.1)
	Totals:	988	450
**Complacency:** Vaccination against HPV is unnecessary because my daughter is not at risk	Strongly disagree	524 (54.0)	139 (31.5)
	Moderately disagree	84 (8.7)	32 (7.3)
	Slightly disagree	55 (5.7)	31 (7.0)
	Neutral	78 (8.0)	95 (21.5)
	Slightly agree	26 (2.7)	27 (6.1)
	Moderately agree	39 (4.0)	35 (7.9)
	Strongly agree	165 (17.0)	82 (18.6)
	Totals:	971	441
**Constraints:** Everyday stress prevents me from getting enough information about HPV vaccination	Strongly disagree	376 (39.4)	130 (29.5)
	Moderately disagree	65 (6.8)	28 (6.4)
	Slightly disagree	67 (7.0)	32 (7.3)
	Neutral	150 (15.7)	103 (23.4)
	Slightly agree	84 (8.8)	36 (8.2)
	Moderately agree	79 (8.3)	45 (10.2)
	Strongly agree	134 (14.0)	67 (15.2)
	Totals:	955	441
**Calculation:** When I think about getting my daughter vaccinated against HPV, I weigh benefits and risks to make the best decision possible	Strongly disagree	157 (16.5)	58 (13.3)
	Moderately disagree	33 (3.5)	23 (5.3)
	Slightly disagree	34 (3.6)	20 (4.6)
	Neutral	120 (12.6)	72 (16.6)
	Slightly agree	63 (6.6)	36 (8.3)
	Moderately agree	116 (12.2)	44 (10.1)
	Strongly agree	431 (45.2)	182 (41.8)
	Totals:	954	435
**Collective responsibility:** When everyone is vaccinated against HPV, I don't have to get my daughter vaccinated too	Strongly disagree	112 (12.0)	67 (15.6)
	Moderately disagree	46 (4.9)	20 (4.7)
	Slightly disagree	20 (2.1)	15 (3.5)
	Neutral	86 (9.2)	84 (19.6)
	Slightly agree	35 (3.7)	24 (5.6)
	Moderately agree	58 (6.2)	31 (7.2)
	Strongly agree	579 (61.9)	188 (43.8)
	Totals:	936	429

**Table 6:** Means of the 5C scores between caregivers of vaccinated and unvaccinated girls and *t*-test *p*-values for statistical significance of difference.

	**Vaccinated** **with ≥1 dose**	**Unvaccinated**	
**Determinant**	**Mean (SD)**	**Mean (SD)**	***P*** **-value**
Confidence	6.2 (1.4)	4.8 (2.2)	0.000
Complacency	2.8 (2.3)	3.6 (2.3)	0.000
Constraints	3.3 (2.3)	3.7 (2.2)	0.003
Calculation	5.1 (2.3)	5.0 (2.2)	0.549
Collective responsibility	5.5 (2.2)	4.9 (2.3)	0.000

## Discussion and Conclusion

### Response Rate

Given that all South African public sector schools providing education to Grade 4 girls, are required by the DBE to participate in the two annual HPV vaccination schools-based campaigns, the relatively low permission rate of 69.6% obtained from school principals was unexpected. However, this permission rate was considerably higher than the 2.3% of South African private sector school principals who had allowed caregivers to participate in a similar survey that we had conducted online in 2018 (1). Of concern is that similar to our previous findings on some private sector school principals (1), some public sector school principals who denied access were clearly either HPV vaccine hesitant or vaccine deniers.

The response rate of 36.8% from caregivers of all girls present on the data collection days, was much higher than the 13.7% response rate from caregivers of age-eligible girls attending South African private sector schools, to the survey we had conducted online in 2018 (1). Of concern is that 17.9% of respondents returned the questionnaires without answering any questions, with 24.8% of them making a statement that they do not give informed consent for their daughters to receive HPV vaccination. This type of negative response, again indicating either HPV vaccine hesitancy or vaccine denial, was reminiscent of the calls made by anti-vaccination lobbyists to boycott our online private sector school HPV vaccination survey when we advertised it on Facebook in 2018 (1).

The implications of these findings will be more fully discussed under limitations to the study.

### HPV Vaccination Coverage

This study was confined to 32 of 46 schools in Sedibeng District with HPV vaccination first dose coverage below 70% in 2018. Thus the finding that only 67.1% of girls in Grades 4 to 7 had received at least one dose of HPV vaccine, with 58.0% being fully vaccinated with both doses, was not unexpected. However, the first dose coverage for Grade 5 girls (who were in Grade 4 in 2018) was found to be 62.2%, which is higher than the official 2018 first dose coverage of 54.2% for Grade 4 girls attending the 32 schools participating in this study. This relatively large discrepancy may be explained by the lack of participation of schools managed by principals who were vaccine hesitant/vaccine deniers, and also the relatively large number of caregivers who either mistook the survey for an informed consent form for HPV vaccination, or used the opportunity to express their rejection of HPV vaccination.

### Reasons for Not Being Vaccinated

It is well-documented that vaccine hesitancy is a major driver of low HPV vaccination uptake globally (2, 7, 8, 18). Thus we were not surprised that almost half the respondents whose daughters were unvaccinated, reported reasons related to vaccine hesitancy, with some giving more than one reason which resulted in almost 60% of the reasons being related to vaccine hesitancy. This finding is supported by our recent report on reasons given by caregivers of unvaccinated age-eligible girls attending private sector schools in South Africa, where 61.4% of reasons were related to vaccine hesitancy (20). In addition, while African data on HPV vaccine hesitancy in caregivers are lacking (2), this finding may explain why obtaining parental informed consent for HPV vaccination was identified as a challenge by stakeholders in six African countries with national school-based HPV vaccination programmes (21).

We had anticipated that reasons related to vaccine hesitancy would feature prominently in our study, since Sedibeng District was affected by anti-vaccination lobbying on social media during the HPV vaccination programme roll-out in 2014, suggesting that this community is susceptible to believing in social media misinformation. In essence, the reasons related to vaccine hesitancy reported by the respondents to our survey, were a reflection of most of the negative sentiments expressed by the public in response to an HPV vaccination-related Facebook post by the Western Cape Department of Health in 2019, six months prior to our survey (8). Our finding that 31.9%, 15.1% and 2.4% of respondents respectively, were concerned about vaccine safety; had heard from “someone” that they or their child had a bad reaction to the HPV vaccine; and had a daughter who had a bad experience with a previous vaccination, resonated with several sentiments reported in the Western Cape study. These include the belief that HPV vaccination was part of “agenda 21” to reduce the population; hearing about people who had suffered serious adverse effects from the HPV vaccine, including death; personally knowing someone who had been permanently adversely affected by the HPV vaccine; personal experience of adverse effects from the HPV vaccine; the perception that parental vaccine safety concerns were being ignored; and the perception that negative safety reports were not in the public domain (8). Also, the perception held by 11.6% of our respondents that their daughters were not at risk for HPV infection, was reported in the Western Cape study as well (8). Finally, the perception that the HPV vaccine is ineffective, reported by 10.5% of our respondents, was also an echo from the Western Cape study, which reported the belief that children receiving HPV vaccination were part of ongoing clinical trials, and there was no proof of the vaccine's effectiveness at preventing cervical cancer (8).

Concerns about the safety of HPV vaccines (or perceived harms of HPV vaccines) has been identified as a major driver of HPV vaccine hesitancy in Europe (22) and the USA (23). Limited data from Africa has also identified HPV vaccine safety concerns in West African countries (24). Similarly, in this study concerns about vaccine safety dominated the reasons related to vaccine hesitancy, with 31.9% of caregivers of unvaccinated girls providing this reason. This finding is supported by our recent report on reasons provided by caregivers of unvaccinated age-eligible girls attending private sector schools in South Africa, where 30.1% were concerned about HPV vaccine safety (20).

Since it has been reported that girls sometimes deliberately don't attend school on HPV vaccination day in order to avoid being vaccinated (19), we had to consider whether or not to incorporate the reason “My daughter was absent from school on vaccination day” as a reason related to vaccine hesitancy. Similarly, the reason “I haven't had time to think about it yet” may be related to vaccine hesitancy, as it has been reported that some healthcare providers erroneously create the impression that the HPV vaccine can safely be postponed if the caregiver needs more time to think about it (18). In the end we classified these two reasons as related to vaccine hesitancy, only when the same respondents had given other reasons related to vaccine hesitancy. Thus it is possible that we may have underestimated the proportion of respondents who gave reasons related to vaccine hesitancy, and this will be further discussed under limitations to the study.

The finding that almost 47% of respondents whose daughters were unvaccinated, were not aware that there was an HPV vaccination programme at their daughter's school, was highly concerning. While this reason for being unvaccinated was reported by respondents with daughters at 28 of the 32 schools, 7 schools were more affected than the others, having ≥10 girls being unvaccinated because of this reason. This may be an indication of gaps in the communication strategy being used for relaying information about the programme to caregivers. The ISHP strategy relies on the relevant class teachers distributing information packages to the girls in their class, who are expected to take these home and give them to their caregivers, and then return the signed informed consent forms to their class teachers before vaccination day (3). Research conducted in Australia (19), which also has a schools-based HPV vaccination programme, provides very helpful information that we can use to improve our national HPV vaccination programme. Caregivers reported that their children may not give them the information, or may not return the signed informed consent forms to their teachers. It was suggested that information should be emailed to caregivers; however, some caregivers did not support this approach. Also, some caregivers suggested that the informed consent process should be completed during enrolment at the beginning of the year, and not “sprung on us”. However, other caregivers rejected this idea (19). These findings suggest that multiple communication strategies should be employed in order to reach high HPV vaccination coverage among age-eligible girls in South Africa.

### Determinants of Vaccine Hesitancy

We had opted to use the 5C scale for investigating vaccine hesitancy, based on the findings from a Western Cape study illustrating the suitability of this model for investigating vaccine hesitancy in South Africa (8). We found that 4 of the 5 constructs (confidence, complacency, constraints and collective responsibility) were very useful for predicting HPV vaccination uptake, with respondents whose daughters were unvaccinated scoring statistically significantly higher for complacency and constraints, while respondents whose daughters were vaccinated scored statistically significantly higher for confidence and collective responsibility. The calculation construct was not a useful predictor, since there was little difference between the unvaccinated and vaccinated. Since high levels of vaccination information-seeking is associated with non-vaccination (9), a higher calculation score in the unvaccinated group was expected. This finding suggests that the phrasing of the calculation statement used in our study (i.e. When I think about getting my daughter vaccinated, I weigh benefits and risks to make the best decision possible) may not be suitable for measuring calculation in the South African context.

### Study Limitations

This study has important limitations that must be considered when interpreting the results. First, while we endeavored to include all schools with <70% first dose HPV vaccination coverage, we were unsuccessful. Furthermore, at least 5 of the 14 schools that were not permitted to participate, were managed by principals who may have been either HPV vaccine hesitant, or HPV vaccine deniers. Three of these principals did not want to upset the majority of caregivers of girls attending their schools, who they described as being opposed to HPV vaccination. Since school principals are highly influential role models for their staff and the broader community, and the success of schools-based HPV vaccination programmes is significantly affected by the attitudes of school teachers (25), these excluded schools may have lower HPV vaccination coverage and higher levels of vaccine hesitancy, than the schools where permission was granted.

Second, the relatively low response rate from caregivers has impacted negatively on the representativeness of this survey, by introducing volunteer bias. The direction of this bias may be explained by the relatively large proportion of questionnaires being returned with all questions being unanswered, including a relatively large number of caregivers using the opportunity to express their opposition to giving consent for their daughters to receive HPV vaccination. It is thus reasonable to suggest that volunteer bias may have resulted in an overestimation of HPV vaccination coverage, and an underestimation of vaccine hesitancy. The former assumption is supported by our finding that the first dose HPV vaccination coverage of Grade 5 girls, is considerably higher than the official coverage reported for 2018, the year that these girls were vaccinated.

Finally, this study may have been subject to recall bias, especially for caregivers of girls who were in Grades 6 and 7, who had been offered HPV vaccination while in grade 4 in 2017 and 2016 respectively. These caregivers had reported a higher ≥1 dose HPV vaccine coverage by their daughters (72.4% and 71.6% respectively) than caregivers of girls in Grades 4 and 5 (64.7% and 62.7% respectively). However, the average official first dose HPV vaccine coverage for 2017 was 77.8% (personal communication, ISHP) compared to 72.4% reported by caregivers of Grade 6 girls in this study, while that for 2016 was 82.6% compared to 71.6% for Grade 7 girls in this study. This indicates that recall bias may have resulted in an underestimation of HPV vaccination coverage of older girls.

### Conclusion and Recommendations

This study is the first South African study to report results of the 5C scale, which was used to measure HPV vaccine hesitancy, with 4 of the 5 constructs found to be very useful for predicting vaccination uptake. It is also the first report confirming that the relatively low HPV vaccination coverage in Sedibeng District is largely driven by reasons related to vaccine hesitancy. What is clear from our results on reasons, is that the ISHP information packages are either not reaching all caregivers, or the language used in these information packages is not well-understood by all caregivers. This does not only pertain to the caregivers who reported being unaware of the HPV vaccination programme; it may also pertain to those reporting reasons related to vaccine hesitancy. Caregivers who do not fully understand the HPV vaccination information supplied by the ISHP may be less likely to allow their daughters to be vaccinated (19), and may subsequently rely on what they have seen on social media to inform their decisions. They may also actively seek information on the internet, where the likelihood of encountering misinformation is high, causing them to make the decision to not vaccinate (8). Our findings suggest the following plan of action:

“*Best practice” and “worst practice” communication strategies used by schools in Sedibeng District need to be identified*. This can be achieved in a relatively short space of time, since official coverage results are reported at school level, and it can safely be assumed that schools with very high coverage are using “best practice” communication strategies. While we have identified 7 schools where “worst practice” strategies are most likely being used, it is safer to assume that all 46 schools with low first dose HPV vaccination coverage may be using “worst practice” communication strategies. In addition, focus group discussions with caregivers at “worst practice” schools should be held in order to identify further specific concerns, information and communication gaps. These data will complement the results of this study, which should be used to update communication packages so that all relevant concerns are addressed. These focus group discussions should also be used to identify the communication strategies preferred by caregivers. Care must be taken to not only adopt the communication strategy preferred by the majority of caregivers, as none should be left uninformed. It could be that a combination of communication strategies will be required, in order to reach all caregivers.

*Develop a standard operating procedure (SOP) for HPV vaccination communication, and mandate all public sector schools where HPV vaccination is offered, to follow this SOP*. This SOP should be based on an analysis of the “best practice” and “worst practice” communication strategies, and educators (including school principals) at all schools should receive training on how best to implement the SOP. While the focus of this training is capacity building of educators to promote HPV vaccination with confidence, it should also improve their role as advocates for public sector school health research and all health interventions offered through the ISHP.

*Conduct research to validate the 5C scale for South Africa*. It is imperative to evaluate the success of the implementation of the “best practice” communication strategy intervention. This can easily be conducted by comparing pre- and post-intervention HPV vaccination coverage. However, comparing pre- and post-intervention vaccine hesitancy levels with a validated 5C tool will provide much-needed data that can be used to identify and address the drivers of vaccine hesitancy beyond the HPV vaccination programme. When conducting this future research, a concerted effort must be made to reach all caregivers and improve the response rate. The possibility that girls may not be taking ISHP information packages home to their caregivers as discussed above, may also have applied to the questionnaires for this survey, providing a further explanation for the low response rate. Although a much lower response rate was achieved in our online private sector survey (1), a combination of emailed and hardcopy questionnaires may produce a higher overall response rate.

## Data Availability Statement

The raw data supporting the conclusions of this article will be made available by the authors, without undue reservation.

## Ethics Statement

The studies involving human participants were reviewed and approved by Sefako Makgatho University Research Ethics Committee of Sefako Makgatho Health Sciences University, Pretoria, South Africa. The participants accepted a consent statement to participate in this study.

## Author Contributions

RB is the grant holder for the over-arching project under which this study falls, and is the co-supervisor of LK. JM is the primary supervisor of LK. JM and CD are co-investigators in the over-arching project, and together with RB developed the grant proposal. FM provided ISHP data and assisted with identifying and accessing schools. Under the supervision of JM and RB, LK wrote the research proposal, obtained ethics clearance, recruited, trained research assistants, obtained permissions, conducted data collection, data analysis, and dissertation writing. All authors gave input into the manuscript, and take equal responsibility for the contents of the manuscript. All authors contributed to the article and approved the submitted version.

## Funding

This study was funded by the National Research Foundation of South Africa (Reference: CPRR150701122450; Grant No: 98959).

## Conflict of Interest

The South African Vaccination and Immunisation Centre, and the Network for Education and Support in Immunisation receive unrestricted educational grants from the vaccine industry. JM declares speaker/consultancy honoraria from the vaccine industry. FM is employed by the National Department of Health. The remaining authors declare that the research was conducted in the absence of any commercial or financial relationships that could be construed as a potential conflict of interest.

## Publisher's Note

All claims expressed in this article are solely those of the authors and do not necessarily represent those of their affiliated organizations, or those of the publisher, the editors and the reviewers. Any product that may be evaluated in this article, or claim that may be made by its manufacturer, is not guaranteed or endorsed by the publisher.
