# Supplementary material for: Vaccine Hesitancy Drives Low Human Papillomavirus Vaccination Coverage in Girls Attending Public Schools in South Africa
Source: Front Public Health. 2022 May 24;10:860809. doi: 10.3389/fpubh.2022.860809 (PMC9171038; doi:10.3389/fpubh.2022.860809)
Supplement: Supplementary file 1 [file Data_Sheet_1.PDF]

frontiers

Supplementary file 1

English questionnaire

Participant number: …………………. **Date: …………………..…**

Title of the survey: Investigation of parental human papillomavirus vaccination decision making amongst caregivers of Grade 4 to 7 girls attending public schools in the Sedibeng District of Gauteng in 2019.

Thank you for taking time to participate in this survey. Read through this page carefully, before proceeding with the questions. The questionnaire itself consists of one and a half pages and will take no longer than 10 minutes to complete. Do not hesitate to contact the researcher (Angela: 078 828 8954) to clarify any uncertainties before completing the survey.

The aim of this survey is to investigate the HPV vaccination decision-making amongst caregivers of Grade 4 to 7 girls attending public schools in the Sedibeng District of Gauteng. The objectives of the study are to i) determine the proportion of girls who received the first dose and girls who received second dose of the HPV vaccine; ii) investigate reasons why caregivers give consent or refuse HPV vaccination; and iii) measure the level of vaccine hesitancy in caregivers of Grade 4 to 7 girls attending public schools in the Sedibeng District of Gauteng.

Consent statement for participation in a Research Project.

By completing this questionnaire, I acknowledge that I have read and understand the above aim and objectives of the survey, and that they are clear to me. I have not been pressurised to participate in any way and I understand that participation in this survey is completely voluntary and that I may withdraw from it at any time and without supplying reasons. I know that this study has received ethical clearance from Sefako Makgatho University Research Ethics Committee (Reference number: SMUREC/P/104/2019: PG). I am fully aware that the results of this study will be used for scientific purposes and may be published. I agree to this, provided my privacy is guaranteed. By completing this questionnaire, I hereby give informed consent to participate in this survey.

Please complete the following questions by placing a CROSS "X" in the relevant block or provide the information requested under "Other". Also provide written answers where necessary.

**Demographic information: Caregiver**					
Date:					
Age of caregiver					
Gender of caregiver	Female			Male	
Race of caregiver	African			White	
	Coloured			Indian or Asian	
	Other (specify):				
Residential area (township/suburb)	Evaton,	Sebokeng		Sharpevile	
	Boipatong	Bophelong		Debonairpark	
	Roshnee	Sonlandpark		Duncanville	
	Arcon Park	Steel Park		Unitas Park	
	Bonanne	Waaldrft		Rust-er-vaal	
	Tshepiso	Other (specify):			
Religion of caregiver					
Caregiver relationship with child	Mother			Grandparent	
	Father			Sibling	
	Other(specify):				
Marital status of the caregiver	Single	Widowed		Living with partner	
	Married	Divorced		Lobola	
Educational level of primary caregiver	No education	Primary school completed		Primary school not completed	
	Secondary school not completed	Secondary school completed		Tertiary Education	
Employment status	Permanently employed	Self-employed	Unemployed	Retired	Disabled, unable to work

Demographic information: Girl child				
Date of birth				
Race	African	White	Other (specify):	
	Coloured	Indian or Asian		
School name				
Grade in school	Grade 4	Grade 5	Grade 6	Grade 7

HPV vaccination questions			
Has your daughter been vaccinated with the HPV vaccine before?	Yes	No	
If yes, which dose of the HPV vaccine did she receive?	1st	2nd	Both
If yes, where did she receive the vaccine?	At school	At the hospital	
	At the clinic	At a campaign	
If at school, in which grade was she when she received HPV vaccination?			

If your daughter has been vaccinated with the HPV vaccine, what are your reasons for giving consent? (please select all reasons that apply)	My family doctor recommended HPV vaccination for her	
	I believe that the HPV vaccine is beneficial for her future health	
	I want to protect her from getting cervical cancer	
	I received reliable and trustworthy information about the HPV vaccine	
	I believe that all vaccines provided by government programmes are safe and effective	
	Other (specify):	
If your daughter has not been vaccinated, what are the reasons for her not receiving the vaccine? (please select all reasons that apply)	I am not aware of a HPV vaccination programme at school	
	The nurses never came to the school to vaccinate the girls	
	My daughter was absent from school on vaccination day	
	My religion prohibits vaccination (please specify the religion)	
	My daughter is not at risk of cervical cancer	
	I haven't had the chance to think about it yet	
	I am concerned about side effects and the safety of this vaccine	
	Someone told me they or their child had a bad reaction to the HPV vaccine	
	I do not think that the HPV vaccine is effective	
	I have no faith in vaccinations in general	
	My daughter has a fear of needles	
	My daughter had a bad experience with a previous vaccination or a vaccinator	
	My healthcare provider has advised me against the HPV vaccine	
	My child has an illness that doesn't allow for vaccinations (please specify the illness)	
	Other (specify):	

Who/what influenced the above decisions?	Friends and family	Social media	Colleagues
	The internet	Nurse advice at clinic	Child's teacher
	Other (specify):		

Do you believe that vaccines can protect children from serious diseases?	Yes	No
Do you think HPV vaccination is necessary for young girls?	Yes	No
Have you ever refused any other vaccination for any of your children?	Yes	No

<table>
<tr><td colspan="4">Have you ever been reluctant or hesitated to get any of your children vaccinated with the HPV or any other vaccine, but then later allowed them to receive the vaccine?</td><td colspan="3">Yes (specify which vaccine/s)</td><td>No</td></tr>
<tr><td colspan="6">Would you advise other parents to get their daughters vaccinated with the HPV vaccine?</td><td>Yes</td><td>No</td></tr>
<tr><td>If not, why?</td><td colspan="7"></td></tr>
<tr><td colspan="8">Please evaluate how much you disagree or agree with the following statements by putting a cross (X) on one of the options in the table below: (1 = strongly disagree, 2 = moderately disagree, 3 = slightly disagree, 4 = neutral, 5 = slightly agree, 6 = moderately agree, 7 = strongly agree)</td></tr>
<tr><td></td><td>1</td><td>2</td><td>3</td><td>4</td><td>5</td><td>6</td><td>7</td></tr>
<tr><td>I am completely confident that the HPV vaccine is safe</td><td>Strongly disagree</td><td>Moderately disagree</td><td>Slightly disagree</td><td>Neutral</td><td>Slightly agree</td><td>Moderately agree</td><td>Strongly agree</td></tr>
<tr><td>Vaccination against HPV is unnecessary because my daughter is not at risk</td><td>Strongly disagree</td><td>Moderately disagree</td><td>Slightly disagree</td><td>Neutral</td><td>Slightly agree</td><td>Moderately agree</td><td>Strongly agree</td></tr>
<tr><td>Everyday stress prevents me from getting enough information about HPV vaccination</td><td>Strongly disagree</td><td>Moderately disagree</td><td>Slightly disagree</td><td>Neutral</td><td>Slightly agree</td><td>Moderately agree</td><td>Strongly agree</td></tr>
<tr><td>When I think about getting my daughter vaccinated, I weigh benefits and risks to make the best decision possible</td><td>Strongly disagree</td><td>Moderately disagree</td><td>Slightly disagree</td><td>Neutral</td><td>Slightly agree</td><td>Moderately agree</td><td>Strongly agree</td></tr>
<tr><td>When everyone is vaccinated against HPV, I don't have to get my daughter vaccinated, too.</td><td>Strongly disagree</td><td>Moderately disagree</td><td>Slightly disagree</td><td>Neutral</td><td>Slightly agree</td><td>Moderately agree</td><td>Strongly agree</td></tr>
</table>

Sesotho questionnaire

Nomoro ea barupelloa:…………………… **Letsatsi:**………….……..

Sehlooho sa phuputso: Phuputso ea ho etsa liqeto tsa ho thibela tšoaetso ea batsoali ba papillomavirus har'a bahlokomeli ba banana ba sehlopha sa bone (4) ho isa ho ba sa (7) ba kenang likolong tsa sechaba setsing sa Sedibeng sa Gauteng ka 2019.

Kea le leboha ka ho ipha nako ea ho kenya letsoho phuputsong ena. Bala leqephe lena ka hloko, pele o tsoela pele ka lipotso. Potso eo ka bo yona e na le maqephe a 'ngoe le halofo' me e tla nka metsotso e fetang 10 ho qeta. U se ke ua tsilatsila ho ikopanya le mofuputsi (Angela: 078 828 8954) ho hlakisa leha ho sa tsitsang pele ho qetoa phuputso.

Sepheo sa phuputso ena ke ho batlisisa ho etsa liqeto ho tsoa ho bahlokomeli ba banana ba likhaolo tsa 4 ho isa ho tse 7 ho ea sekolong sa Sedibeng sa Gauteng. Sepheo sa thuto ke ho (i) ho lekanyetsa palo ea banana ba fumaneng tekano ea pele le banana ba fumaneng leano la bobeli la ente ea HPV; ii) batlisisa mabaka a etsang hore bahlokomeli ba fane ka tumello kapa ba hane ho entoa ka HPV; le iii) lekanya hore na thibelo ea ente e tsilatsile joang ho bahlokomeli ba banana ba 4 ho isa ho ba 7 ba kenang likolong tsa sechaba setsing sa Sedibeng sa Gauteng.

Polelo ea tumello ea ho kenya letsoho Phatlalatsong ea Lipatlisiso

Ka ho tlatsa lipotso tsena, kea lumela hore ke balile le ho utloisisa sepheo se ka holimo le tsa phuputso, le hore li hlakile ho 'na. Ha kea qobelloa hore nka kopanela ka tsela e le efe 'me ke utloisisa hore ho kenya letsoho phuputsong ena ke ka boithatelo le hore nka tlohela ka nako efe le ntle le ho fana ka mabaka. Kea tseba hore thuto ena e amohetse tumello ea molao ho tsoa Sefako Makgatho University Research Research Ethics Committee (Reference Reference: SMUREC/P/104/2019: PG). Ke tseba hantle hore qeto ea thuto ena li tla sebelisoa ka morero oa saense mme li ka phatlalatsoa. Ke lumellana le sena, ha feela sephiri sa ka se tiisitsoe. Ka ho tlatsa lipotso tsena, ke fana ka tumello e nepahetseng ea ho kopanela phuputsong ena.

Ka kopo, tlatsa lipotso tse latelang ka ho beha CROSS "X" lebokoseng le nepahetseng kapa fana ka boitsebiso bo botsoang tlas'a "Tse ling". Hape fana ka likarabo tse ngotsoeng moo ho hlokahalang.

Boitsebiso ba palo ea batho: Mohlokomeli					
Letsasi:					
Lilemo tsa mohlokomeli					
Mohlokomeli ke	Mosadi	Monna			
Lebala la mohlokomeli	African	White			
	Coloured	Indian kapa Asian			
	Tse ling (Hlakisa):				
Sebaka sa bolulo	Evaton,	Sebokeng	Sharpevile		
	Boipatong	Bophelong	Debonairpark		
	Roshnee	Sonlandpark	Duncanville		
	Arcon Park	Steel Park	Unitas Park		
	Bonanne	Waaldrft	Rust-er-vaal		
	Tshepiso	Meyerton			
	Other (hlakisa)				
Bolumeli ba tlhokomelo					
Kamano ea mohlokomeli le ngoana	Mme	Mme e moholo/ Ntate-moholo			
	Ntate	Ngoan'eso			
	Other (hlakisa):				
Boemo ba lenyalo ba mohlokomeli	Ke masoha	Ke Mohlolohali	Ke dula le molekane		
	Ke nyetsoe	Ke hlalile	Lobola		
Lekala la thuto la tlhokomelo ea mantlha	Ha ho thuto	Sekolo sa mathomo	Sekolo se bohareng		
	Sekolo sa sekolo se sa phethoa	Sekolo sa Sekondari	Thuto e phahameng		
Sebaka sa mosebetsi	Kea sebetsa	Kea itšebetsa	Ha ke sebetse	ke tlohetse mosebetsi	ke na le bokooa, ke sitoa ho sebetsa

<table>
<tr><th colspan="8">Boitsebiso ba palo ea batho: Ngoana oa ngoananyana</th></tr>
<tr><td>Letsatsi la tsoalo</td><td colspan="7"></td></tr>
<tr><td rowspan="2">Lebala</td><td colspan="3">African</td><td colspan="4">White</td></tr>
<tr><td colspan="3">Coloured</td><td colspan="4">Indian or Asian</td></tr>
<tr><td></td><td colspan="7">Tse lingwe (hlakisa):</td></tr>
<tr><td>Lebitso la sekolo</td><td colspan="7"></td></tr>
<tr><td>Sehlopha sekolong</td><td>Grade 4</td><td>Grade 5</td><td>Grade 6</td><td colspan="4">Grade 7</td></tr>
<tr><th colspan="8">Lipotso tsa thibelo ea HPV (ente ea HPV)</th></tr>
<tr><td colspan="4">Na morali oa hao o 'nile a entoa ka ente ea HPV pele?</td><td>E.</td><td colspan="3">Che</td></tr>
<tr><td colspan="4">Haeba e, o na le tekanyo efe ea thibelo ea HPV?</td><td>Es mathomo</td><td>Ea bo beli</td><td colspan="2">Ka bobeli</td></tr>
<tr><td colspan="4" rowspan="2">Haeba Karabo ke e, o e fumane ho kae?</td><td>At school</td><td colspan="3">Sepetlele</td></tr>
<tr><td>Kliniki</td><td colspan="3">Nakong ea letšolo</td></tr>
<tr><td colspan="4">Haeba a le sekolong, o ne a le sehlopheng sefe ha a fuoa thibelo ea HPV?</td><td colspan="4"></td></tr>
<tr><td rowspan="6">Haeba morali oa hao a entsuoe ka ente ea thibelo ea HPV, ke hobane'ng ha u fana ka tumello? (ka kopo, khetha mabaka 'ohle a sebetsang)</td><td colspan="6">Ngaka ea lelapa e ile ea khothalletsa thibelo ea HPV bakeng sa eona</td><td></td></tr>
<tr><td colspan="6">Ke lumela hore thibelo ea HPV e molemo bakeng sa bophelo bo botle ba nako e tlang</td><td></td></tr>
<tr><td colspan="6">Ke batla ho mo sireletsa hore a se ke a tšoaroa ke kankere ea malapa</td><td></td></tr>
<tr><td colspan="6">Ke fumane tlhahisoleseling e tšepahalang le e ka tšeptjoang ka thibelo ea HPV</td><td></td></tr>
<tr><td colspan="6">Ke lumela hore liente tsohle tse fanoang ke mananeo a mmuso li sireletsehile ebile lia sebetsa</td><td></td></tr>
<tr><td colspan="7">Tse ling (hlakisa):</td></tr>
<tr><td rowspan="14">Haeba morali oa hao a e-s'o ka a entoa, ke mabaka afe a sa mo amoheleng?</td><td colspan="6">Ha ke tsebe lenaneho la ho thibela HPV sekolong</td><td></td></tr>
<tr><td colspan="6">Baoki ha baa ka ba tla sekolong ho ea finyella banana</td><td></td></tr>
<tr><td colspan="6">Morali oa ka o ne a le sieo sekolong ka letsatsi la ho entoa</td><td></td></tr>
<tr><td colspan="6">Bolumeli ba ka bo thibela ho entoa (ka kopo, hlalosa bolumeli)
____________________</td><td></td></tr>
<tr><td colspan="6">Morali oa ka ha a kotsing ea lefu la kankere ea malapa</td><td></td></tr>
<tr><td colspan="6">Ha ke e-s'o bele monyetla oa ho nahana ka eona leha ho le joalo</td><td></td></tr>
<tr><td colspan="6">Ke amehile ka litla-morao le polokeho ea ente ena</td><td></td></tr>
<tr><td colspan="6">Motho e mong o ile a mpolella hore bona kapa bana ba bona ba na le maikutlo a fosahetseng ho thibelo ea HPV</td><td></td></tr>
<tr><td colspan="6">Ha ke nahane hore ente ea HPV e atleha</td><td></td></tr>
<tr><td colspan="6">Ha ke na tumelo ho liente ka kakaretso</td><td></td></tr>
<tr><td colspan="6">Morali oa ka o tšaba lisebelisoa</td><td></td></tr>
<tr><td colspan="6">Morali oa ka o ne a e-na le boiphihlelo bo bobe ka ente e entsoeng ka pele ho nako kapa ka vaccinator</td><td></td></tr>
<tr><td colspan="6">Mofani oa ka oa tlhokomelo ea bophelo o nkhothalelitse khahlanong le ente ea HPV</td><td></td></tr>
<tr><td colspan="6">Ngoan'a ka o na le boloetse bo sa lumelleheng bakeng sa liente (ka kopo o hlalose boloetse)
____________________</td><td></td></tr>
<tr><td></td><td colspan="7">Tse ling (hlakisa):
____________________</td></tr>
<tr><td rowspan="3">Ke mang / eng e ileng ea susumetsa liqeto tse holimo?</td><td colspan="2">Metsoalle ea ka le ba lelapa</td><td colspan="2">Social media</td><td colspan="3">Basebetsi-'moho</td></tr>
<tr><td colspan="2">Internet</td><td colspan="2">Mooki keletso ho tliliniki</td><td colspan="3">Tichere ea ngoana</td></tr>
<tr><td colspan="7">Tse lingwe (hlakisa):</td></tr>
</table>

Na u lumela hore liente li ka sireletsa bana ho mafu a tebileng?	E.	Che
Na u nahana hore thibelo ea HPV e hlokahala bakeng sa banana ba banyenyane?	E.	Che
Na o kile oa hana ente efe kapa efe ea bana ba hau?	E.	Che
Na u kile ua tsilatsila kapa oa tsilatsila ho fumana bana ba hau ba nang le HPV kapa ente leha e le efe, empa hamorao ba ba lumella hore ba fumane ente ee?	E.	Che
Na u ka eletsa batsoali ba bang ho fumanela bana ba bona ente ea HPV?	E.	Che
Haeba ha ho joalo, hobane'ng?		

Ka kopo lekola hore na ha u lumellane hakae kapa u lumellana le polelo e latelang: 1= hana haholo, 2= hana ka tekano 3= hanetsana hanyane 4= se nke lehlakore 5= lumela hanyane 6= lumela ka tekano 7= lumela haholo.							
	1	2	3	4	5	6	7
Ke tshepetse ka botlalo hore ente ea HPV e bolokehile.	Hana haholo	Hana ka tekano	Hanetsana hanyane	Se nke lehlakore	Lumela hanyane	Lumela ka tekano	Lumela haholo
Ente khahlanong le HPV ha e hlokahale hobane morali oa ka ha a kotsing.	Hana haholo	Hana ka tekano	Hanetsana hanyane	Se nke lehlakore	Lumela hanyane	Lumela ka tekano	Lumela haholo
Matshoenyeho a letsatsi le letsatsi a nthibela ho fumana tlhaiso-leseling e lekaneng ka thibelo ea HPV.	Hana haholo	Hana ka tekano	Hanetsana hanyane	Se nke lehlakore	Lumela hanyane	Lumela ka tekano	Lumela haholo
Ha ke nahana ka ho thibela morali oa ka hore a enteloe, ke nahana ka melemo le likotsi tsa ho etsa qeto e ntle ka ho fetisisa.	Hana haholo	Hana ka tekano	Hanetsana hanyane	Se nke lehlakore	Lumela hanyane	Lumela ka tekano	Lumela haholo
Ha motho e mong le e mong a entetsoe HPV, ha ke a tlameha ho fumana le morali oa ka a ente	Hana haholo	Hana ka tekano	Hanetsana hanyane	Se nke lehlakore	Lumela hanyane	Lumela ka tekano	Lumela haholo

Afrikaans questionnaire

Deelnemer vraelys (Afrikaans)

Vraelys verwysingsnommer: ………………….　　　　　　**Datum: ……………………..…**

Titel van die Opname: Navorsing oor die besluitnemingsproses van ouers/versorgers van Graad 4 tot 7 dogters wat publieke skole bywoon in die Sedibeng Distrik van die Gauteng Provinsie, aangaande die menslike papilloma virus (HPV) inenting, gedurende 2019.

Byvoorbaat dankie vir u tyd en bereidwilligheid om deel te neem aan hierdie opname. Lees asseblief hierdie bladsy sorgvuldig, voordat u die vraelys begin voltooi. Die vraelys is een en 'n half bladsy lank en sal nie meer as 10 minute neem om te voltooi nie. Indien u enige vrae het, kontak asb. die navorser (Angela: 078 828 8954) om enige onsekerhede op te klaar voordat u die vraelys voltooi.

Die doel van die opname is om te bepaal watter faktore die besluitnemingsproses beinvloed van ouers/versorgers van Graad 4 tot 7 dogters wat publieke skole bywoon aangaande HPV inenting, Verder gaan i) bepaal word watter persentasie dogters reeds die eerste en tweede dosering van die HPV inenting ontvang het; ii) redes ondersoek word waarom versorgers toestemming gegee het vir HPV inenting, of waarom hulle nie toestemming gegee het nie; iii) bepaal word tot watter mate entstof huiwering ervaar word deur versorgers van Graad 4 tot 7 dogters, wat publieke skole bywoon.

Toestemming verklaring van deelnemers aan die Navorsings Projek / Opname

Deur die voltooiing van die vraelys, erken ek dat ek die doel van die navorsing gelees en verstaan het, en dat dit duidelik was vir my. Ek was geensins onder druk geplaas om deel te neem aan die navorsingsprojek nie. Ek verstaan dat my deelname vrywillig is, en dat ek enige tyd my deelname kan ontrek, sonder om 'n verduideliking daarvoor te verskaf. Ek verstaan dat die navorsingsprojek etiese klaring ontvang het vanaf 'Sefako Makgatho University Research Ethics Committee' (Reference number: SMUREC/P/104/2019: PG) en is bewus dat die resultate van die navorsing vir wetenskaplike doeleindes gebruik sal word en moontlik gepubliseer kan word. Ek stem daarmee in, met dien verstande dat my privaatheid ten alle tye gewaarborg sal word. Deur die voltooiing van die vraelys gee ek hiermee toestemming vir deelname aan die opname.

Voltooi asseblief die volgende vraelys deur 'n kruisie te plaas in die bepaalde blokkie soos voorsien is, of verskaf die nodige inligting in die blokkie gemerk "Ander". Voorsien asseblief ook geskrewe antwoorde of verduidelikings waar nodig.

<table>
<tr><th colspan="6">Demografiese inligting van ouers/versorgers</th></tr>
<tr><td>Datum:</td><td colspan="5"></td></tr>
<tr><td>Ouderdom van versorger:</td><td colspan="5"></td></tr>
<tr><td>Geslag van versorger:</td><td colspan="3">Vroulik</td><td colspan="2">Manlik</td></tr>
<tr><td rowspan="3">Ras van versorger</td><td colspan="3">Swart</td><td colspan="2">Wit</td></tr>
<tr><td colspan="3">Kleurling</td><td colspan="2">Indiër / Asiër</td></tr>
<tr><td colspan="5">Ander (spesifiseer):</td></tr>
<tr><td rowspan="6">Residentsiële area (Dorp/Uitbreiding)</td><td colspan="2">Evaton</td><td colspan="2">Sebokeng</td><td>Sharpevile</td></tr>
<tr><td colspan="2">Boipatong</td><td colspan="2">Bophelong</td><td>Debonairpark</td></tr>
<tr><td colspan="2">Roshnee</td><td colspan="2">Sonlandpark</td><td>Duncanville</td></tr>
<tr><td colspan="2">Arcon Park</td><td colspan="2">Steel Park</td><td>Unitas Park</td></tr>
<tr><td colspan="2">Bonanne</td><td colspan="2">Waaldrft</td><td>Rust-er-vaal</td></tr>
<tr><td colspan="2">Tshepiso</td><td colspan="3">Other (spesifiseer):</td></tr>
<tr><td>Voorkeur godsdiens van versorger</td><td colspan="5"></td></tr>
<tr><td rowspan="3">Verwantskap van versorger tot die kind</td><td colspan="3">Moeder</td><td colspan="2">Grootouer</td></tr>
<tr><td colspan="3">Vader</td><td colspan="2">Broer/Suster</td></tr>
<tr><td colspan="5">Ander (spesifiseer):</td></tr>
<tr><td rowspan="2">Huweliks status van versorger</td><td colspan="2">Ongetroud</td><td colspan="2">Weduwee/Wewenaar</td><td>Woon saam venoot</td></tr>
<tr><td colspan="2">Getroud</td><td colspan="2">Geskei</td><td>Lobola</td></tr>
<tr><td rowspan="2">Opvoeding vlak van versorger</td><td colspan="2">Geen opvoeding</td><td colspan="2">Laerskool voltooi</td><td>Laerskool nie voltooi</td></tr>
<tr><td colspan="2">Hoërskool nie voltooi</td><td colspan="2">Hoërskool voltooi</td><td>Tersiëre opvoeding</td></tr>
<tr><td>Status van indiensnemings</td><td>Vas aangestel</td><td>Self-werksaam</td><td>Werkloos</td><td>Afgetree</td><td>Ongeskik om te werk</td></tr>
</table>

frontiers

**Demografiese inligting van dogter**			
Geboortedatum			
Rassegroep	Swart	Wit / Blank	Ander (spesifiseer):
	Kleurling	Indiër / Asiër	
Naam van skool			
Graad van leerling	Graad 5	Graad 6	Graad 7

**HPV inventings vrealys**			
Was u dogter vantevore ingeënt met die HPV vaksien?	Ja	Nee	
Indien <u>JA</u>, watter dosering van die HPV vaksien het sy ontvang?	1ste	2de	Albei
Indien <u>JA</u>, waar het sy die vaksien ontvang?	By die skool	By die hospitaal	
	By die kliniek	Tydens ‘n veldtog	
Indien by die skool, in watter graad was sy, toe sy die HPV inenting ontvang het?			

Indien u dogter REEDS ingeënt is met die HPV vaksien, waarom het u daarvoor toestemming gegee? (Merk asb. al die redes wat van toepassing is)	My huisdokter het die HPV inenting vir haar aanbeveel	
	Ek glo die HPV inenting sal voordelig wees vir haar toekomstige gesondheid	
	Ek wil haar beskerm daarteen om servikale kanker op te doen	
	Ek het betroubare inligting aangaande die HPV intenting ontvang	
	Ek glo dat alle intentings wat deur die staatsprogam voorsien word veilig en effektief is	
	Ander redes (verduidelik):	
Indien u dogter nog NIE ingeënt is met die HPV vaksien NIE, waarom het sy dit nie ontvang nie? (Merk asb. al die redes wat van toepassing is)	Ek is nie bewus van die HPV inentings program by die skool nie	
	Die verpleegsusters het nie na die skool gekom om die dogters in te ent nie.	
	My dogter was afwesig die dag waarop die inentings plaasgevind het	
	My geloof verbied inenting (Dui asb die godsdiens aan)	
	My dogter is nie in gevaar om servikale kanker te kry nie	
	Ek het nog nie tyd gehad om daaroor te dink nie	
	Ek is bekommered oor die newe effekte en die veiligheid van die vaksien	
	Iemand het vir my vertel dat hulle kind ‘n slegte reaksie met die HPV vaksien gehad het	
	Ek dink nie dat die HPV vaksien veilig is nie	
	Ek glo in die algemeen nie aan inentings nie	
	My dogter is bang vir naalde	
	My dogter het ‘n slegte ervaring gehad met ‘n vorige inenting of met die persoon wat die inenting toegedien het	
	My gesondheidsorg diensverskaffer het my afgeraai (nie aanbeveel) dat sy die HPV inenting kry	
	My kind ly aan ‘n mediese toestand wat intenging nie toelaat nie (Dui asb. die mediese toestand aan)	
	Ander redes (verduidelik):	

Wie/wat het die bogenoemde besluit/e beïnvloed?	Vriende en familie	Sosiale media	Kollegas
	Die internet	Advies van die verpleegsuster by die kliniek	Kind se onderwyser
	Ander (verduidelik):		

Glo u dat inenting u kind kan beskerm teen ernstige siektes?	Ja	Nee
Glo u dat die HPV inenting nodig is vir jong meisies?	Ja	Nee
Het u al ooit geweier dat u kind enige ander vaksiene kan ontvang?	Ja	Nee

Was u al ooit huiwerig om enige van u kinders te laat inent met die HPV vaksien, of enige ander vaksien, maar het later wel besluit om hulle te laat intent?	Ja (watter vaksien/vaksiene)	Nee
Sal u ander versorgers / ouers aanbeveel om hulle dogters te laat inent met die HPV vaksien?	Ja	Nee
Indien NIE, verduidelik asb. waarom nie.		

Evalueer asseblief die volgende stellings, en dui aan tot watter mate u daarmee saamstem, of nie saamstem nie. Maak 'n kruisie in die blokkie van u keuse: (1 = Stem sterk nie saam nie, 2 = Stem matig nie saam nie, 3 = Stem gedeeltelik nie saam nie, 4 = neutraal, 5 = Stem gedeeltelik saam, 6 = Stem matig saam, 7 = Stem sterk saam)							
	1	2	3	4	5	6	7
Ek is vol vertroue dat die HPV inenting veilig is.	Stem sterk nie saam nie	Stem matig nie saam nie	Stem gedeeltelik nie saam nie	Neutraal	Stem gedeeltelik saam	Stem matig saam	Stem sterk saam
Inenting teen HPV is onnodig want my dogter is nie in gevaar daarvoor nie.	Stem sterk nie saam nie	Stem matig nie saam nie	Stem gedeeltelik nie saam nie	Neutraal	Stem gedeeltelik saam	Stem matig saam	Stem sterk saam
Daaglikse bekommernisse verhoed my om genoeg inligting van HPV inentings te kry.	Stem sterk nie saam nie	Stem matig nie saam nie	Stem gedeeltelik nie saam nie	Neutraal	Stem gedeeltelik saam	Stem matig saam	Stem sterk saam
Wanneer ek daaroor dink om my dogter te laat inent, oorweeg ek die voordele en nadele om die beste besluit te maak.	Stem sterk nie saam nie	Stem matig nie saam nie	Stem gedeeltelik nie saam nie	Neutraal	Stem gedeeltelik saam	Stem matig saam	Stem sterk saam
Wanneer almal anders ingeënt is teen HPV, hoef ek nie my dogter in te ent nie.	Stem sterk nie saam nie	Stem matig nie saam nie	Stem gedeeltelik nie saam nie	Neutraal	Stem gedeeltelik saam	Stem matig saam	Stem sterk saam

BAIE DANKIE VIR U DEELNAME EN TYD!
